# Dendritic spine density of prefrontal layer 6 pyramidal neurons in relation to apical dendrite sculpting by nicotinic acetylcholine receptors

**DOI:** 10.3389/fncel.2015.00398

**Published:** 2015-10-08

**Authors:** Lily Kang, Michael K. Tian, Craig D. C. Bailey, Evelyn K. Lambe

**Affiliations:** ^1^Department of Physiology, University of TorontoToronto, ON, Canada; ^2^Department of Biomedical Sciences, Ontario Veterinary College, University of GuelphGuelph, ON, Canada; ^3^Department of Obstetrics and Gynecology, University of TorontoToronto, ON, Canada

**Keywords:** prefrontal cortex, layer 6, dendritic spines, nicotinic acetylcholine receptors, chrna5, nicotine, mouse, morphology

## Abstract

Prefrontal layer 6 (L6) pyramidal neurons play an important role in the adult control of attention, facilitated by their strong activation by nicotinic acetylcholine receptors. These neurons in mouse association cortex are distinctive morphologically when compared to L6 neurons in primary cortical regions. Roughly equal proportions of the prefrontal L6 neurons have apical dendrites that are “long” (reaching to the pial surface) vs. “short” (terminating in the deep layers, as in primary cortical regions). This distinct prefrontal morphological pattern is established in the post-juvenile period and appears dependent on nicotinic receptors. Here, we examine dendritic spine densities in these two subgroups of prefrontal L6 pyramidal neurons under control conditions as well as after perturbation of nicotinic acetylcholine receptors. In control mice, the long neurons have significantly greater apical and basal dendritic spine density compared to the short neurons. Furthermore, manipulations of nicotinic receptors (*chrna5* deletion or chronic developmental nicotine exposure) have distinct effects on these two subgroups of L6 neurons: apical spine density is significantly reduced in long neurons, and basal spine density is significantly increased in short neurons. These changes appear dependent on the α5 nicotinic subunit encoded by *chrna5*. Overall, the two subgroups of prefrontal L6 neurons appear positioned to integrate information either across cortex (long neurons) or within the deep layers (short neurons), and nicotinic perturbations differently alter spine density within each subgroup.

## Introduction

Layer 6 (L6) pyramidal neurons exert complex effects on higher cognitive processes through “top-down” thalamic feedback (Zhang and Deschênes, 1998; Alitto and Usrey, 2003; Lam and Sherman, 2010; Thomson, 2010) and through direct control over cortical excitation (Olsen et al., 2012; Kim et al., 2014). In particular, L6 neurons in medial prefrontal cortex (Bailey et al., 2010) and their strong excitation by nicotinic acetylcholine receptors (Parikh et al., 2007) are essential for optimal performance on attention tasks (Bailey et al., 2010; Guillem et al., 2011). Prefrontal L6 pyramidal neurons receive a much stronger nicotinic excitation than those in other cortical regions (Tian et al., 2014), and when compared to neurons in other layers of prefrontal cortex (Poorthuis et al., 2013). These neurons receive cholinergic afferents from the basal forebrain (Woolf, 1991; Proulx et al., 2014), and express high affinity nicotinic acetylcholine receptors with relevant subunits detected on their somata and proximal apical dendrites (Bailey et al., 2012; Proulx et al., 2014).

Acetylcholine and agonists of nicotinic receptors are also known morphogens, with powerful effects on dendritic arborization (Pugh and Berg, 1994; Ballesteros-Yáñez et al., 2010; Bailey et al., 2012, 2014; Mychasiuk et al., 2013) and dendritic spine density (Brown and Kolb, 2001; Ballesteros-Yáñez et al., 2010; Lozada et al., 2012; Mychasiuk et al., 2013). Yet, the normal spine density and the consequences of nicotinic perturbations for spine density on prefrontal L6 pyramidal neurons have not yet been examined. Morphological studies examining the typical density and localization of dendritic spines on layer 6 neurons have focused exclusively on primary sensory and motor cortices (Konur et al., 2003; Gao and Zheng, 2004; Orner et al., 2013). These studies report that L6 cells have sparse dendritic spines compared to neurons of other cortical layers (Konur et al., 2003; Gao and Zheng, 2004) and these spines undergo substantial pruning and shape changes during post-natal development (Orner et al., 2013). The effects of nicotinic perturbation on L6 spine density are unknown. Studies have investigated the consequences of nicotinic receptor perturbations on spine density in the other layers of prefrontal cortex (Brown and Kolb, 2001; Ballesteros-Yáñez et al., 2010; Mychasiuk et al., 2013). In cortical layers not strongly stimulated by nicotinic receptors, nicotinic perturbations had variable consequences for spine density: nicotine exposure led to an increase in spine density (Brown and Kolb, 2001; Mychasiuk et al., 2013) and loss of the β2 nicotinic subunit to a decrease (Ballesteros-Yáñez et al., 2010). These other pyramidal neurons are thought to receive primarily indirect effects of nicotinic stimulation (Lambe et al., 2003, 2005; Poorthuis et al., 2013), in contrast to the direct nicotinic stimulation of L6.

Here, we examine dendritic spine density on L6 pyramidal neurons that receive strong, direct nicotinic stimulation in a region of association cortex essential for attention (Muir et al., 1996; Dalley et al., 2004). Prefrontal L6 neurons stand out from L6 pyramidal neurons in other cortical regions for an unusual post-juvenile developmental pattern. Unlike primary cortex L6 cells which predominantly terminate in the deeper cortical layers during post-natal development and in adulthood (Zhang and Deschênes, 1998; Brumberg et al., 2003; Zarrinpar and Callaway, 2006; Chen et al., 2009), L6 neurons in medial prefrontal cortex have a majority of “long” neurons with apical dendrites stretching across the cortex to the pial surface in the juvenile period (Bailey et al., 2012, 2014). In the post-juvenile period in mice, a nicotinic receptor dependent mechanism leads half of the L6 neurons to develop the “short” phenotype of apical dendrites typical of L6 neurons in other cortical regions, while the other half retain their “long” apical dendrite (Bailey et al., 2012, 2014). Manipulations of nicotinic receptors have been shown to alter this normal post-juvenile change (Bailey et al., 2012, 2014). However, the spine density and localization profiles are unknown in both subpopulations of prefrontal L6 neurons, as are the effects of nicotinic manipulations on these measures. Investigation of nicotinic receptor dependent changes in dendritic spines on these L6 neurons can offer insight into various cognitive disorders. Perturbations in nicotinic receptors alter attention (Bailey et al., 2010; Guillem et al., 2011), cognition (Ernst et al., 2001; Granon et al., 2003), anxiety and social behavior in adulthood (Vaglenova et al., 2004; Ekblad et al., 2010; Chabout et al., 2013), all of which involve the integration of complex information by the prefrontal cortex.

## Methods

### Experimental animals and study design

Dendritic spine density analysis was performed on neurons collected for two previous studies (Bailey et al., 2012, 2014). In brief, male adult C57BL/6 mice (age: P60–150; mean ± SE: 106 ± 26 days; *n* = 30) in this work were either homozygous wildtype (α5^+∕+^) or homozygous null (α5^−∕−^; α5 knockout; α5 KO) for the α5 nicotinic acetylcholine receptor subunit (Salas et al., 2003). These mice were not more than two generations descended from heterozygous (α5^+∕−^) crosses, and a subset of the mice had knockin α4-YFP nicotinic receptor subunits, which permitted *post-hoc* laminar confirmation with immunohistochemistry (Bailey et al., 2012, 2014). For the chronic nicotine exposure, a subgroup of wildtype and α5 KO mice was treated with nicotine throughout gestation and through development up to post-natal day 21 (randomly assigned pregnant females and then their pups were given drinking water with either 200 μg/mL nicotine tartrate and 2% saccharin (wt/vol) or tartaric acid vehicle and 2% saccharin). Care was given to limit both animal suffering and the quantity of animals used. This protocol was approved by the University of Toronto Animal Care Committee and follows the rules and regulations of the Canadian Council on Animal Care.

The methods to fill, record from, and collect the neurons have been reported previously (Bailey et al., 2012, 2014). In brief, 400 μm coronal brain slices of mPFC (Bregma 1.98–1.18 mm; Paxinos and Franklin, 2001) were prepared. L6 pyramidal neurons were patched in the whole-cell configuration with a pipette filled with 0.3% neurobiotin. Pyramidal neurons were selected based on their characteristic shape using IR-DIC visualization. Neurons were patched in the mid- to deep-portions of layer 6, and this laminar placement was confirmed with electrophysiological examination of nicotinic acetylcholine currents. In a subset of cases, additional laminar confirmation was obtained histologically, as described above. For the electrophysiological experiments, the peak magnitude of the nicotinic acetylcholine response was assessed in voltage clamp at a holding potential of −75 mV with brief bath application of acetylcholine (1 mM) in the presence of atropine (200 nM) to block muscarinic receptors. Afterwards, the brain slices were fixed overnight with 4% (wt/vol) paraformaldehyde, reacted with streptavidin conjugated to the fluorophore Alexa-594, and cover-slipped. Multiphoton imaging was acquired using a Ti:sapphire laser with a wavelength of 780 nm and a Olympus Fluoview FV1000 microscope with an Olympus XLPlan N 25X, 1.05 NA water-immersion objective. Imaging with 2x Kalman sampling captured slightly overlapping 3D stacks of each fluorescent L6 pyramidal neuron, including all basal and apical dendritic branches. These stacks were stitched together using Neurolucida software and all dendrites traced for morphology analysis. Correction for tissue shrinkage was not applied; however, the Z thickness of the fixed slices suggests it is on the order of ~50%, which is not unexpected given the fixative used (Wehrl et al., 2015). To give the maximal power to the analysis of L6 neurons with “long” vs. “short” apical dendrites, as well as the comparison of long neurons and short neurons by genotype, data from two studies (Bailey et al., 2012, 2014) were used together. This combined dataset retained the significant increase (Fisher's exact test, *p* < 0.05) in the proportion of L6 neurons with long apical dendrites in the α5 knockout group: 71% “long” (Long: *n* = 20; Total: *n* = 28), compared to the 46% “long” seen in wildtype (Long: *n* = 17; Total: *n* = 37).

### Dendritic spine density quantification and analysis

**Figure 1:**
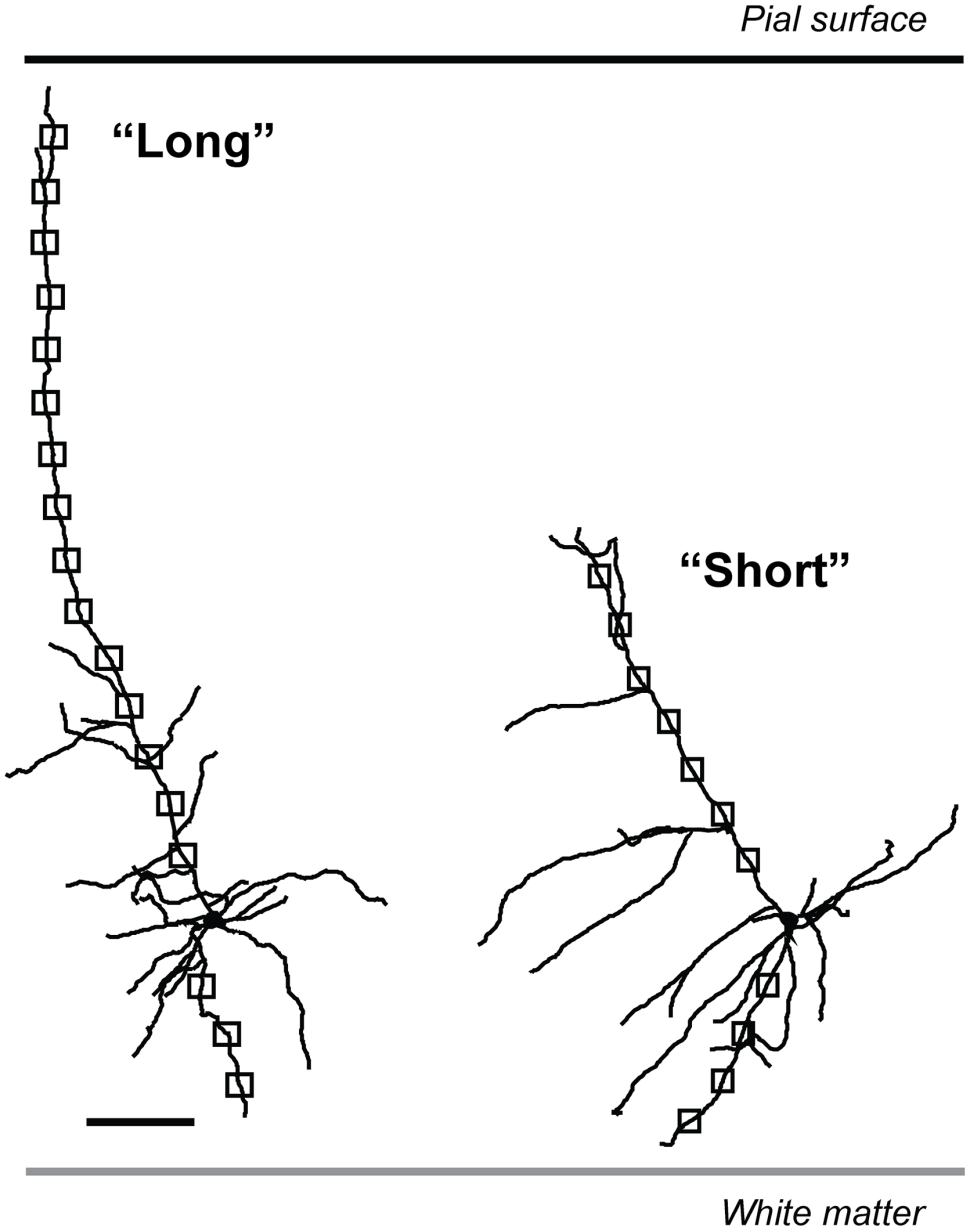
**Tracings of representative “long” and “short” layer 6 pyramidal neurons in mouse medial prefrontal cortex to demonstrate dendritic spine density sampling areas**. The regions of dendritic spine sampling are illustrated with boxes: 20 μm segments beginning at each Sholl intersection point from the soma to the ends of each respective dendrite type. Scale bar, 100 μm. Error bars plotted for SEM.

All analysis was performed blind to genotyping and treatment group. The spine analysis was done using both Neurolucida and Neurolucida Explorer software (MBF Bioscience, Williston, VT, USA). A schematic illustrating the areas of dendritic spine sampling for all neurons is shown in Figure 1. At each Sholl intersection point (50 μm) (Sholl, 1953), all dendritic spines were counted for 20 μm along the main apical dendrite and one basal dendrite. Since initial sampling showed that spine densities across basal dendrites within a neuron did not differ significantly [*F*_(4, 32)_ = 0.3, *p* = 0.9, Two-Way ANOVA], the longest basal dendrite from each neuron was chosen for analysis. In addition, the longest possible extension of the apical dendrite was analyzed. As the apical dendrite diverged into the apical tuft, the branch that was closest to the pial surface was chosen for the remainder of the spine analysis sampling. Dendritic spine data was analyzed by Two-Way ANOVA and the F-ratios are shown with the degrees of freedom of tested groups in parentheses. For all statistical analysis, we used a significance level of *p* < 0.05. These analyses were conducted with GraphPad Prism 5 (GraphPad Software, La Jolla, CA, USA).

## Results

### Differences in dendritic spine density on “long” and “short” L6 neurons

**Figure 2:**
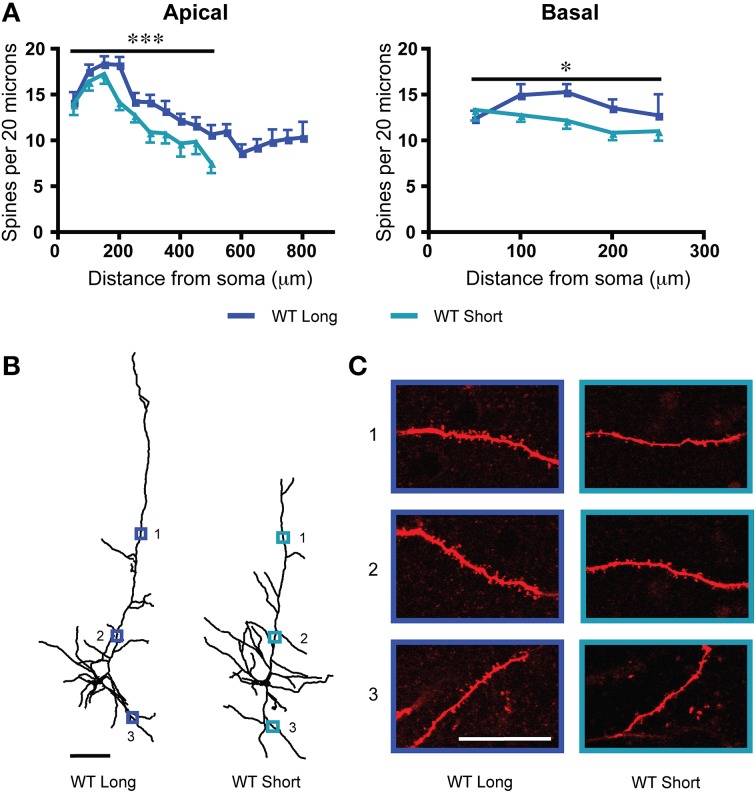
**In wildtype (WT) mice, there are significant differences in dendritic spine density between the long and short layer 6 pyramidal neurons. (A)** Long neurons have significantly greater spine densities compared to short neurons on both apical (^***^p < 0.0001, Two-Way ANOVA) and basal (^*^p = 0.02, Two-Way ANOVA) dendrites. **(B)** Representative tracings from each subgroup. Scale bar, 100 μm. **(C)** Higher magnification images for illustrative purposes from the apical and basal dendrites of these neurons, respective regions denoted by numbered boxes (note: images rotated for convenience of comparison). Scale bar, 20 μm. Color code: dark blue, WT long neurons; light blue, WT short neurons. Error bars plotted for SEM.

We sampled dendritic spine density from two subgroups of L6 neurons from wildtype mice: “long” neurons with apical dendrites that reached across to the pial surface and “short” neurons with apical dendrites that terminated at or before the mid-layer of cortex (Bailey et al., 2012, 2014). As shown in Figure 2A, long neurons had significantly greater dendritic spine density along their apical dendrites [*F*_(1, 283)_ = 18.9, *p* < 0.0001, Two-Way ANOVA] as well as along their basal dendrites [*F*_(1, 130)_ = 5.5, *p* = 0.02, Two-Way ANOVA]. To illustrate these differences in dendritic spine density between long and short L6 neurons, examples from each subtype are also shown in Figures 2B,C. For the apical dendrites, there were also significant effects of position along the dendrite in both subtypes of neurons [*F*_(9, 283)_ = 13.9, *p* < 0.0001, Two-Way ANOVA], with distal apical dendrites having lower dendritic spine densities. However, the electrophysiological response to nicotinic acetylcholine stimulation showed no difference between these two subgroups in adulthood (long neurons: wildtype: 80.9 ± 8.6 pA; short neurons: wildtype: 68.8 ± 8.1 pA; *t*_31_ = 1.0, *p* = 0.3, *t*-test).

### Effects of nicotinic acetylcholine manipulations on L6 dendritic spine density

The morphogenic effects of acetylcholine and the presence of nicotinic acetylcholine receptors in L6 pyramidal neurons suggest a potential mechanism for the regulation of their dendritic spine density. To test this hypothesis, we examined changes in dendritic spine densities of long and short L6 neurons following manipulation of nicotinic acetylcholine receptors by either deletion of the α5 subunit, or chronic exposure to nicotine *in vivo*. Either of these manipulations impairs the nicotinic receptor-mediated effects of acetylcholine on L6 pyramidal neurons (Bailey et al., 2010, 2012, 2014).

**Figure 3:**
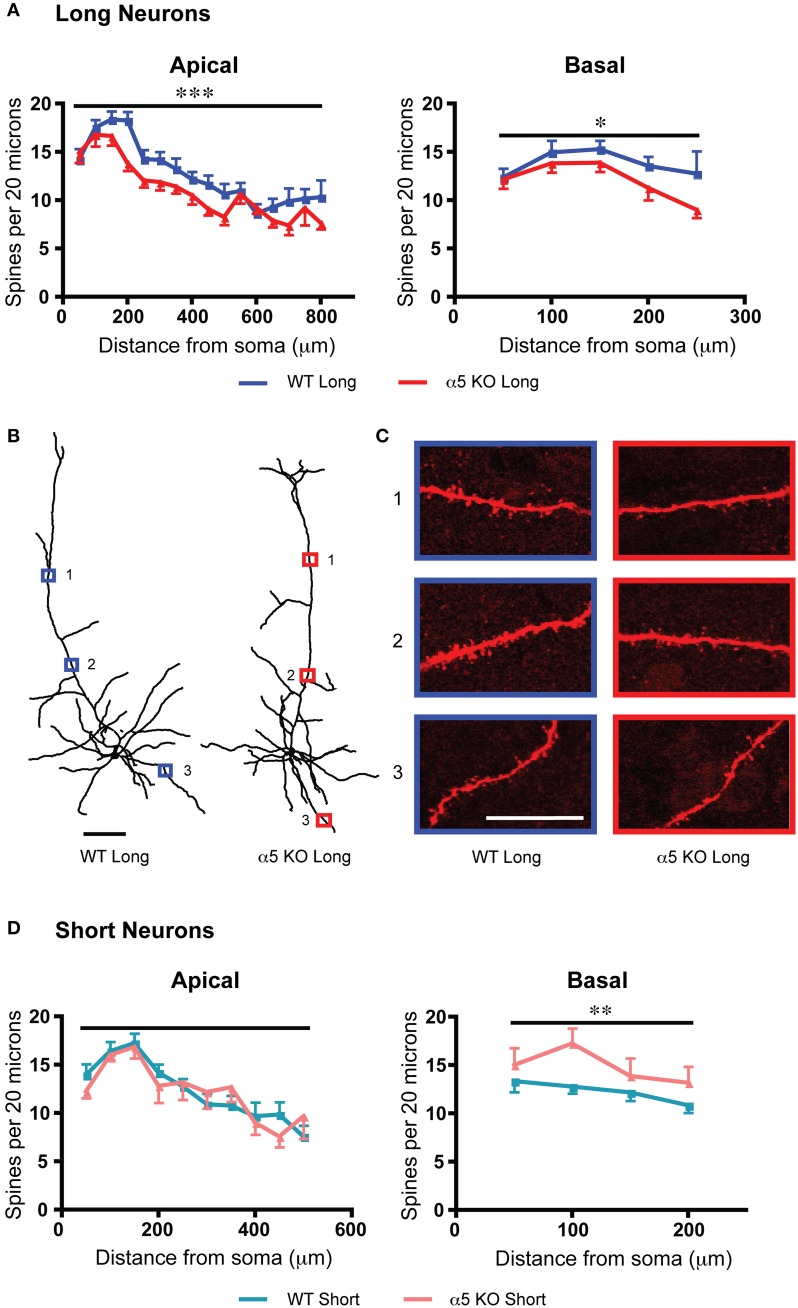
**For long and short layer 6 neurons, deletion of the α5 nicotinic subunit (α5 knockout; α5 KO) results in a different pattern of spine density changes compared to wildtype (WT) mice. (A)** For long neurons, dendritic spine density was significantly decreased by α5 KO on both apical (^***^p < 0.0001, Two-Way ANOVA) and basal (^*^p = 0.04, Two-Way ANOVA) dendrites, compared to WT neurons. **(B)** Representative tracings of long neurons from each genotype. Scale bar, 100 μm. **(C)** Higher magnification images for illustrative purposes from the apical and basal dendrites of these neurons, respective regions denoted by numbered boxes (note: images rotated for convenience of comparison). Scale bar, 20 μm. **(D)** By contrast, short neurons showed no significant effect of α5 KO genotype on apical dendritic spine density (*p* = 0.8, Two-Way ANOVA). For short neurons, α5 KO significantly increased basal dendritic spine density compared to WT (^**^p = 0.007, Two-Way ANOVA). Color code: dark blue, WT long neurons; red, α5 KO long neurons; light blue, WT short neurons; orange, α5 KO short neurons. Error bars plotted for SEM.

The α5 nicotinic receptor subunit is expressed in prefrontal L6 (Wada et al., 1990) and contributes to normal L6 responses to acetylcholine and optimal attentional performance (Bailey et al., 2010). Its deletion reduces the nicotinic effects of acetylcholine in L6 pyramidal neurons (Bailey et al., 2010, 2012, 2014). In long neurons, deletion of the α5 nicotinic subunit resulted in a significant decrease in apical dendritic spine density [*F*_(1, 451)_ = 17.2, *p* < 0.0001, Two-Way ANOVA] and a more modest decrease in basal dendritic spine density [*F*_(1, 119)_ = 4.5, *p* = 0.04, Two-Way ANOVA]. For short neurons, by contrast, α5 knockout resulted in a significant increase in the basal dendritic spine density [*F*_(1, 92)_ = 7.6, *p* = 0.007, Two-Way ANOVA] and no significant difference in the apical spine density. These changes are illustrated in Figure 3. Both groups showed significant effects of position along the apical dendrite on spine density [long: *F*_(15, 451)_ = 19.2, *p* < 0.0001; short: *F*_(9, 173)_ = 8.9, *p* < 0.0001, Two-Way ANOVA]. Furthermore, α5 deletion was accompanied by significantly decreased electrophysiological responses to nicotinic stimulation in both long and short neurons compared to control mice (long: wildtype: 80.9 ± 8.6 pA; α5 knockout: 54.0 ± 4.9 pA; *t*_32_ = 2.8, *p* = 0.009, *t*-test; short: wildtype: 68.8 ± 8.1 pA; α5 knockout: 37.3 ± 2.2 pA; *t*_21_ = 2.3, *p* = 0.03, *t*-test).

**Figure 4:**
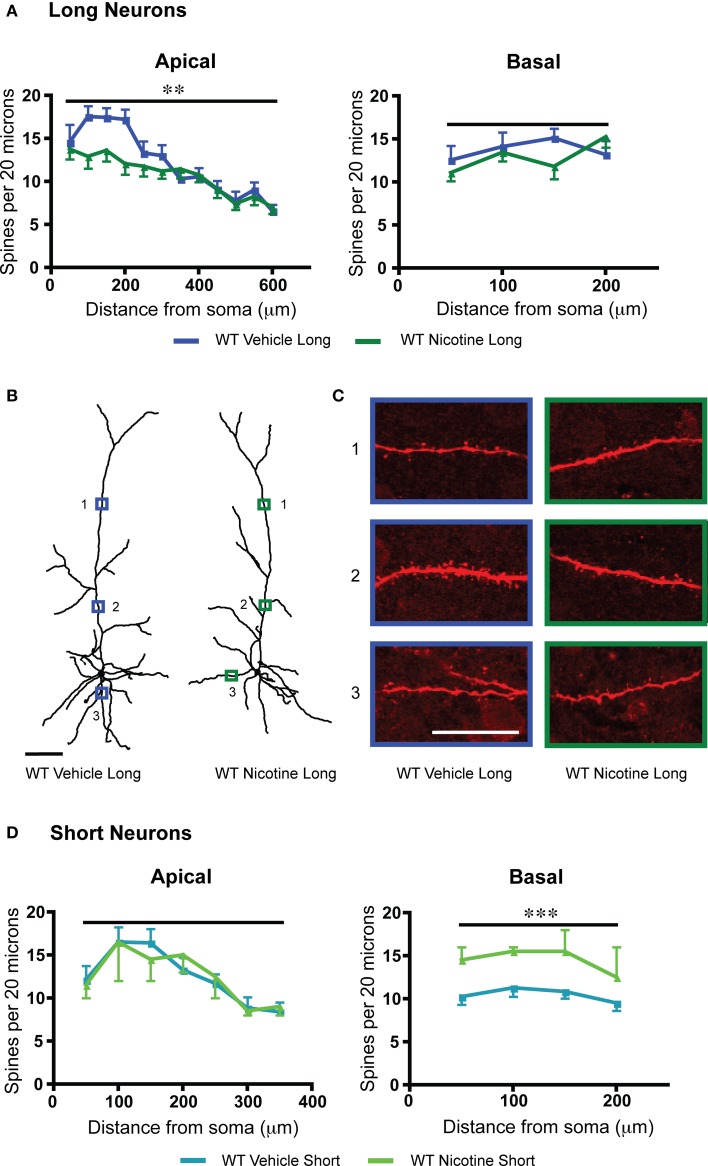
**Within each subgroup of layer 6 neurons of wildtype (WT) mice, chronic nicotine results in a different pattern of spine density changes compared to vehicle control. (A)** For long neurons, apical dendritic spine density was significantly reduced (^**^p < 0.003, Two-Way ANOVA), without altered basal dendritic spine density (*p* = 0.4, Two-Way ANOVA). **(B)** Representative tracings of long neurons from each treatment group. Scale bar, 100 μm. **(C)** Higher magnification images for illustrative purposes from the apical and basal dendrites of these neurons, respective regions denoted by numbered boxes (note: images rotated for convenience of comparison). Scale bar, 20 μm. **(D)** By contrast, short neurons showed no significant effect of chronic nicotine treatment for apical spine density (*p* = 0.98, Two-Way ANOVA). For short neurons, chronic nicotine significantly increased basal dendritic spine density (^***^p = 0.0005, Two-Way ANOVA). Color code: dark blue, vehicle long neurons; dark green, nicotine long neurons; light blue, vehicle short neurons; light green, nicotine short neurons. Error bars plotted for SEM.

Nicotine initially activates but then strongly desensitizes nicotinic acetylcholine receptor currents in L6 prefrontal pyramidal neurons (Bailey et al., 2010, 2014). Chronic exposure leads to a long-lasting reduction of L6 nicotinic excitation in WT mice but not α5 KO mice (Bailey et al., 2014). Therefore, as an alternative method to probe the consequences and mechanisms of reduced nicotinic acetylcholine receptor function, we examined L6 dendritic spine density in WT mice which had been treated throughout development until the juvenile period with 200 μg/mL nicotine tartrate and 2% saccharin (wt/vol) in their drinking water. Compared to vehicle-matched controls, the long neurons in the group with the nicotinic manipulation showed significantly decreased apical spine density [*F*_(1, 230)_ = 8.8, *p* = 0.003, Two-Way ANOVA], and the short neurons showed significantly increased basal spine density [*F*_(1, 28)_ = 15.2, *p* = 0.0005, Two-Way ANOVA], as illustrated in Figure 4. Both long and short neurons showed significant effects of position along the apical dendrite on spine density [long: *F*_(11, 230)_ = 12.9, *p* < 0.0001; short: *F*_(6, 51)_ = 4.0, *p* = 0.003, Two-Way ANOVA]. Significantly lower nicotinic responses were observed electrophysiologically in the wildtype nicotine-treated group compared to wildtype vehicle [long: vehicle: 84.8 ± 13.7 pA, nicotine: 26.7 ± 2.2 pA; *t*_19_ = 4.8, *p* = 0.0001, *t*-test; short: vehicle: 71.4 ± 6.1 pA, nicotine: 31.2 ± 11.4 pA; *t*_8_ = 3.0, *p* = 0.02, *t*-test).

**Figure 5:**
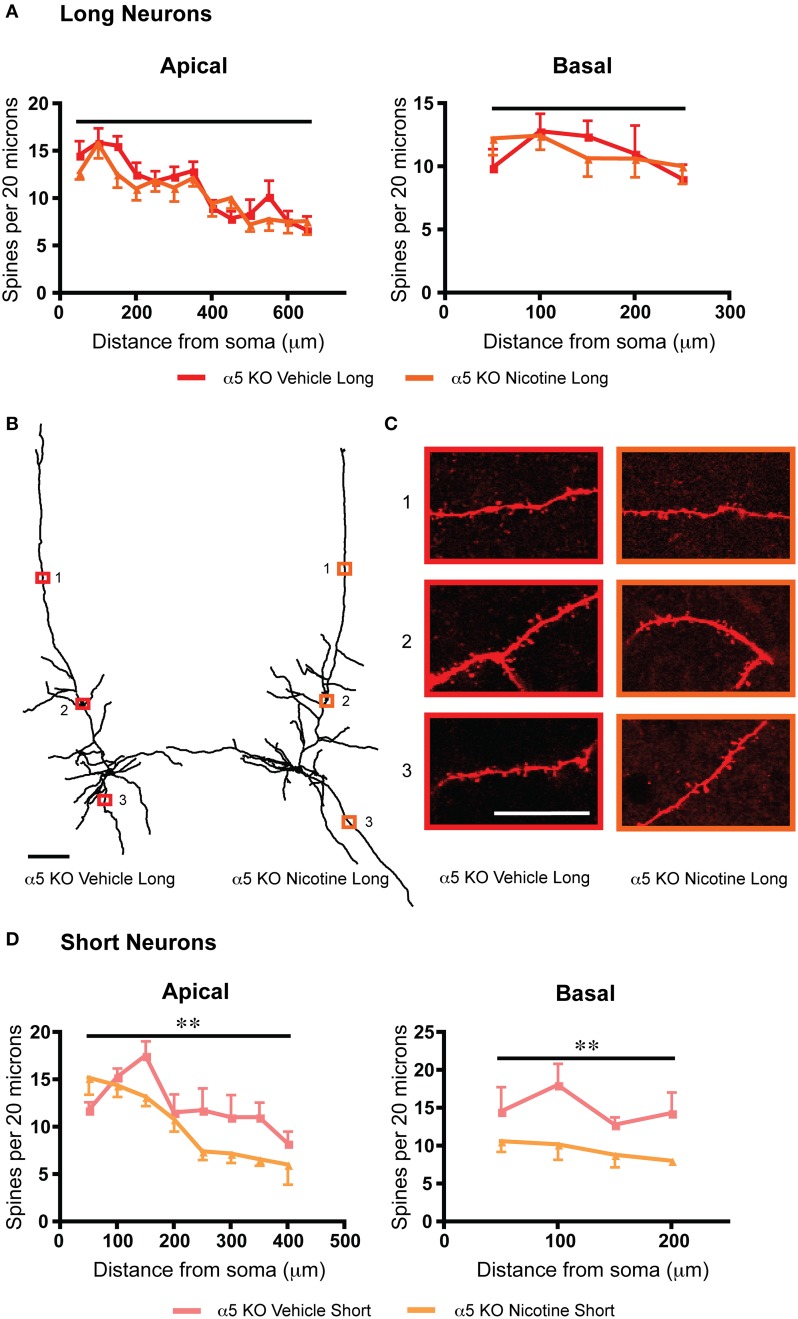
**Deletion of the α5 nicotinic subunit occludes the effects of chronic nicotine on dendritic spine density for long neurons and produces a different pattern of changes for short neurons. (A)** For long neurons, there is no significant difference in spine density between α5 KO vehicle and α5 KO nicotine for either the apical (*p* = 0.2, Two-Way ANOVA) or basal (*p* = 0.9, Two-Way ANOVA) dendrites. **(B)** Representative tracings of long neurons from each group. Scale bar, 100 μm. **(C)** Higher magnification images for illustrative purposes from the apical and basal dendrites of these neurons, respective regions denoted by numbered boxes (note: images rotated for convenience of comparison). Scale bar, 20 μm. **(D)** By contrast, the short neurons of α5 KO mice exposed to nicotine showed a significant decrease in spine density in both the apical (^**^p = 0.005, Two-Way ANOVA) and basal dendrite (^**^p = 0.002, Two-Way ANOVA), compared to vehicle-exposed α5 KO. Color code: red: α5 KO vehicle long; orange, α5 KO nicotine long; light red, α5 KO vehicle short; light orange, α5 KO nicotine short. Error bars plotted for SEM.

To probe whether a shared mechanism underlies the similar effects on dendritic spine density arising from α5 subunit deletion or chronic nicotine treatment, we examined an additional group of α5 knockout mice, which had been exposed chronically to nicotine along with vehicle-matched controls. In these nicotine-treated α5 knockout mice, there was no further significant difference in spine density on the apical dendrites of their long neurons, as illustrated in Figure 5. These results suggest that loss of this nicotinic receptor subunit may occlude the effects of chronic nicotine. Whereas the short α5 knockout neurons showed a different pattern of response to chronic nicotine than wildtype, with significant reductions in both apical [*F*_(1, 54)_ = 8.8, *p* = 0.005, Two-Way ANOVA) and basal dendritic spine density [*F*_(1, 24)_ = 11.7, *p* = 0.002, Two-Way ANOVA]. Of note, the opposite electrophysiological effect was observed for nicotine treatment in the α5 knockout mice (long: α5 knockout with vehicle: 53.7 ± 6.5 pA, α5 knockout with nicotine: 75.2 ± 5.6 pA; *t*_16_ = 2.5, *p* = 0.02, *t*-test; short: α5 knockout with vehicle: 40.1 ± 2.0 pA, α5 knockout with nicotine: 60.4 ± 13.3 pA; *t*_7_ = 1.3, *p* = 0.2, *t*-test).

## Discussion

**Figure 6:**
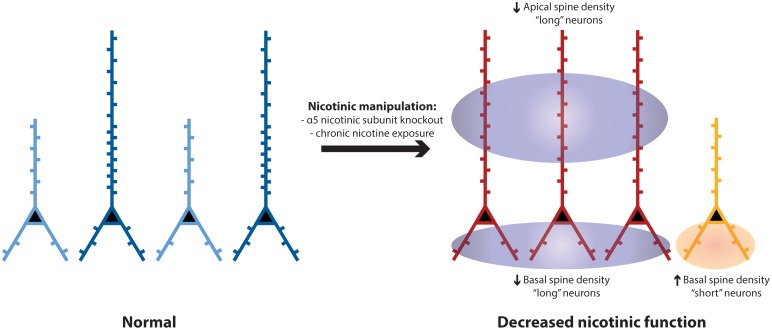
**Schematic to summarize spine density differences between long and short L6 pyramidal neurons in wildtype controls, and the differences in neuronal morphology and pattern of spine density that develop following nicotinic perturbations (e.g., α5 KO)**. In medial prefrontal cortex of wildtype mice, there are similar proportions of “long” layer 6 pyramidal neurons (shown in dark blue, with apical dendrites stretching across the cortical mantle) and “short” ones (light blue, with apical dendrites terminating in the deep or mid-layers of prefrontal cortex) (Bailey et al., 2012, 2014). The long neurons have significantly greater spine density on both their apical and basal dendrites, compared to short neurons. In mice with disruption of nicotinic receptors (such as deletion of the α5 nicotinic receptor subunit or exposure to chronic nicotine), the more-prevalent long neurons (red) have significantly *reduced* apical spine density in adulthood, whereas the less-prevalent short neurons (orange) have significantly *increased* basal spine density in adulthood.

We show that two morphologically-distinct subgroups of prefrontal L6 neurons are significantly different in spine density on their apical and basal dendrites. The long neurons, positioned to integrate information across the entire cortical column, showed greater spine density compared to the short neurons with dendrites that are restricted to the deep layers of cortex. Although both subgroups of L6 neurons typically have robust responses to nicotinic acetylcholine stimulation, each subgroup appears to have a specific pattern of dendritic spine density changes in response to perturbations of nicotinic receptors. As illustrated in Figure 6, apical spine density was significantly *reduced* only in the long neurons; whereas, basal spine density was significantly *increased* only in the short neurons. These changes appear dependent on the α5 nicotinic receptor subunit. Overall, nicotinic perturbations may disrupt or distort the integration of information by prefrontal L6 neurons important in executive function.

Dendritic spines are the primary targets of excitatory glutamatergic input to cortical pyramidal neurons. Spines serve as important biochemical compartments (Yuste et al., 2000) and yet are well-coupled electrically to the dendritic tree (Grunditz et al., 2008; Gulledge et al., 2012; Popovic et al., 2012). Prefrontal L6 pyramidal neurons have sufficiently thin dendrites (Bailey et al., 2014) to achieve an NMDA spike with only the activation of a small numbers of spines (Major et al., 2008, 2013; Polsky et al., 2009). In thin dendrites, NMDA spikes are robust, with a large safety factor (Schiller et al., 2000; Gordon et al., 2006; Branco and Häusser, 2011; Major et al., 2013). Recordings from the apical dendrites of L6 neurons in primary sensory cortex have shown local dendritic spikes from a variety of stimuli, including NMDA spike electrogenesis (Ledergerber and Larkum, 2010). Such L6 apical dendrite electrogenesis allowed the integration of inputs to different layers to influence neuronal output (Ledergerber and Larkum, 2010). In prefrontal cortex, this phenomenon may contribute to the persistent firing (Major and Tank, 2004; Antic et al., 2010) required for executive function.

Medial prefrontal cortex receives glutamatergic afferents from diverse brain regions, including limbic cortical regions (Van Eden et al., 1992), midline and intralaminar thalamus (Berendse and Groenewegen, 1991; Lambe and Aghajanian, 2003), amygdala (Bacon et al., 1996; Gabbott et al., 2012), and hippocampus (Swanson, 1981; Ferino et al., 1987; Jay and Witter, 1991; Carr and Sesack, 1996; Hoover and Vertes, 2007; Parent et al., 2010; Vertes et al., 2015). While these inputs have characteristic patterns of laminar termination, the laminar targeting of the apical and basal dendrites of L6 neurons is not well understood. In prefrontal neurons of other layers, there appears to be a segregation of inputs to the apical and basal dendrites, with basal dendrites receiving inputs from the amygdala that can be activated by stress (Liu et al., 2015) and apical dendrites receiving inputs from the midline thalamus (Lambe and Aghajanian, 2003; Lambe et al., 2005), which may be more cognition or attention-focused (Sarter et al., 2014). Of note, stress in adulthood results in selective reduction in inputs to the apical dendrites, while inputs to the basal dendrites appear resilient to stress (Cook and Wellman, 2004).

Among cortical neurons, prefrontal L6 pyramidal neurons receive unusually strong excitation by nicotinic acetylcholine receptors (Bailey et al., 2010; Poorthuis et al., 2013; Tian et al., 2014; Hedrick and Waters, 2015). Nicotinic receptors can exert morphogenic effects on spine density (Lozada et al., 2012). Indeed, significant effects of nicotinic manipulations on spine density are seen even in neurons of layers and regions that see relatively weak, mainly-indirect nicotinic effects (Brown and Kolb, 2001; Ballesteros-Yáñez et al., 2010; Mychasiuk et al., 2013). In these neuronal populations, nicotinic receptor manipulations did not exert a consistent direction of changes in spine density, with increases in response to nicotine (Brown and Kolb, 2001; Lozada et al., 2012; Mychasiuk et al., 2013) and decreases with deletion of the β2 nicotinic subunit (Ballesteros-Yáñez et al., 2010; Lozada et al., 2012), but the changes were similar across apical and basal dendrites when both were examined (Brown and Kolb, 2001; Mychasiuk et al., 2013). By contrast, manipulation of other neurotransmitter systems has been shown preferentially to perturb one type of dendrites; for example, deletion of certain dopamine receptors decreased spine density only on basal dendrites (Wang et al., 2009) and a model of NMDA hypofunction decreased spine density only on apical dendrites (Balu and Coyle, 2012).

Here, in neurons known to have prominent nicotinic effects on the post-juvenile sculpting of their apical dendrites (Bailey et al., 2012, 2014), we see specific adult differences in dendritic spine density at baseline that are distinct in the two different L6 neuronal subgroups. With abnormal nicotinic receptor function, long neurons that receive information across all the prefrontal laminae show significantly reduced apical dendritic spine density; whereas the short neurons that integrate information within the deep cortical layers show significantly increased basal dendritic spine density. The asymmetric changes to dendritic morphology (Bailey et al., 2012, 2014) and spine density resulting from nicotinic perturbations are illustrated in Figure 6 and hint at complex underlying mechanisms dependent on normal nicotinic function in development. The sensitivity of L6 neurons to nicotinic stimulation during development (Kassam et al., 2008; Bailey et al., 2012) and its consequences for apical dendritic retraction (Bailey et al., 2012, 2014) raise the speculation that the long neurons in mice exposed to nicotinic perturbations simply manifest the lower dendritic spine density expected for the short phenotype. However, other mechanisms may be responsible since dendritic spines on L6 neurons show a prolonged and fluid period of development (Orner et al., 2013) during which they may be particularly sensitive to perturbed excitatory neuromodulation.

Overall, the pattern of changes is noteworthy because a growing body of research suggests that apical and basal dendrites may be the targets of distinct categories of inputs (Lambe et al., 2003, 2005; Gabbott et al., 2012; Liu et al., 2015) and their dendritic spines may be differentially sensitive to stress (Cook and Wellman, 2004). The pattern suggests that nicotinic perturbations may lead to prefrontal attention circuitry that is less nuanced in its integration of information across the layers of cortex and more sensitive to a narrower subset of afferents. The potential interaction between these changes and those resulting from stress is a key subject for future investigation since nicotinic perturbations in humans and preclinical models exacerbate the adverse behavioral consequences of subsequent stress exposure (Ekblad et al., 2010; Nosjean et al., 2014; Park et al., 2014).

## Author contributions

Conceived and designed the experiments: LK, CB, MT, and EL. Performed the experiments and analyzed the data: LK and CB. Wrote the paper: LK, MT, CB, and EL. All authors contributed to revision of the article, and approved the final version of the manuscript.

### Conflict of interest statement

The authors declare that the research was conducted in the absence of any commercial or financial relationships that could be construed as a potential conflict of interest.
